# Eliminating Cervical Cancer in Mali and Senegal, Two Sub-Saharan Countries: Insights and Optimizing Solutions

**DOI:** 10.3390/vaccines8020181

**Published:** 2020-04-14

**Authors:** Azizul Haque, Bourèma Kouriba, N’diaye Aïssatou, Anudeep Pant

**Affiliations:** 1 Department of Microbiology and Immunology, Geisel School of Medicine at Dartmouth, 1 Medical Center Drive, Lebanon, NH 03756, USA; 2 Parasite Cellular and Molecular Immunology Unit, Université des Sciences des Techniques et des Technologies de Bamako, Bamako BP1805, Mali; kouriba@icermali.org; 3 Mid-Wife (Sage—Femme), Centre de santé Philippe Maguiléne Senghor, District Ouest Dakar, Senegal; aissatoumail@yahoo.fr; 4 New Orleans East Hospital, 5620 Read Blvd., New Orleans, LA 70127, USA; Anudeep.Pant@lcmchealth.org

**Keywords:** cervical cancer, Sub-Saharan Africa, HPV, vaccination, screening, risk factors, awareness, prevalence, treatments, health care delivery

## Abstract

Background: The number of cases with cervical cancer is rapidly increasing in Sub-Saharan Africa driven by inadequate rates of human papilloma virus (HPV) vaccination and screening programs and accompanied by poor health delivery systems. There are other factors to contend with such as lack of awareness, social myths, reluctance to vaccine acceptance and stigma with sexually transmitted diseases. Here, we formulate strategies to implement intervention programs against HPV infections and other risk factors for cervical cancer in these countries. Methods: We searched PubMed, Web of Science, and African Journals Online for this review. The current status of anti-HPV vaccination and precancerous screening programs in Mali and Senegal has been assessed by onsite visits. Collaborators from Mali and Senegal collected data and information concerning HPV vaccination and screening programs in these countries. Findings: We found that anti-HPV vaccination and cervical cancer screening have been conducted sporadically mainly in urban areas of Mali and Senegal. No known population-based programs are in progress in either of the two countries. We highlighted the advantages and drawbacks of currently available screening tests and proposed that screening by visual inspection with acetic acid (VIA) accompanied by self-sampling is the most cost-effective, culturally acceptable and most feasible strategy to implement in primary care settings. In addition, HPV DNA testing would be affordable, if local laboratory facilities could be established. We found that many of the factors that increase HPV acquisition and promote the oncogenic effect of the virus are largely widespread in both Senegal and Mali. These include infections with HIV and other sexually transmitted infections (STIs), immunosuppression, polygamous marriages, high parity, early sexual activities, early pregnancies, and multiple sexual partners. Interpretation: Neither vaccines nor screening tests are within the reach of the population in Mali and Senegal because of the high cost. The effective intervention measure would be to integrate anti-HPV vaccines into the Extended Program for Immunization (EPI), which has saved 3 million young lives per year in Africa with the support of GAVI, to implement cost control mechanisms for HPV vaccinations via price negotiations with manufacturing companies, as has recently been done by Rwanda. The collective efforts by local governments, researchers, private sector, and donors may lead to the introduction of affordable screening tests. A robust awareness campaign coupled with sustained and regular engagement of local communities about the prevention and risk factors is extremely important. The projected solutions may be well applicable to other Sub-Saharan countries that face similar challenges containing cervical cancer.

## 1. Introduction

The incidence of cervical cancer has been greatly reduced in high-income countries by the use of human papilloma virus (HPV) vaccines and cervical cancer screenings. However, its incidence is on the rise and is second only to breast cancer in low-income countries, especially in Sub-Sahara [1,2]. Cervical cancer causes estimated 265,672 deaths annually, of which nearly 87% deaths occur in less developed countries [3,4]. This inequality is mainly due to the lack of functional HPV vaccination and screening programs, the cost and absence of coverage, and the lack of infrastructure.

This paper reviews the current status of anti-HPV vaccination and precancerous screening programs in two Sub-Saharan countries, namely Mali and Senegal. We advocate for cost control mechanisms, increased awareness, and the implementation of extended vaccination and screening programs. We formulate strategies to implement intervention programs against HPV infections and other risk factors for cervical cancer in these countries. Despite the current challenges, the proposed strategies present feasible solutions to eliminate cervical cancer in the coming decades from this part of the world.

## 2. Pathogenesis of HPV in Cervical Cancer

**Figure 1:**
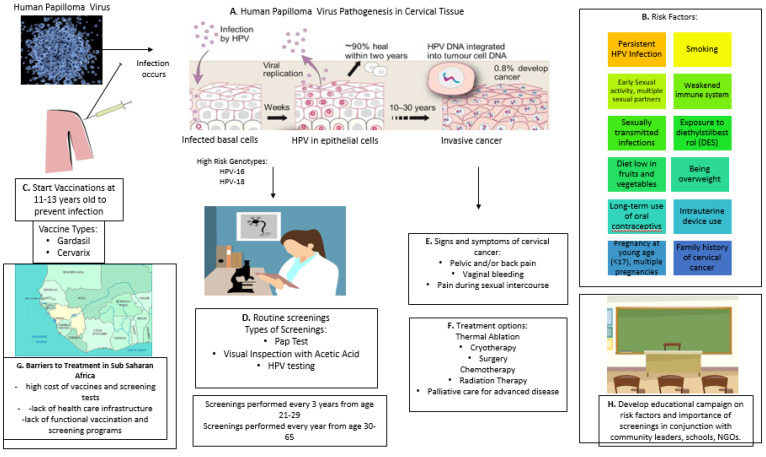
Cervical cancer pathogenesis, role of human papilloma virus (HPV), impact of vaccination, symptoms and potential treatments, and risk factors for cervical cancer development. (**A**). Human Papilloma Virus (HPV) Pathogenesis, (**B**). Risk factors, (**C**). HPV vaccinations, (**D**). Routine screening and types of tests, (**E**). Signs and symptoms of cervical cancer, (**F**). Treatment options, (**G**). Barriers to containment of cervical cancer, (**H**). Developing educational campaign.

HPV is a highly infectious virus transmitted through oral, anal, or genital sexual contact, as well as through nonpenetrative sex involving skin-to-skin contact [5]. It is estimated that 80% of sexually active women will have at least one HPV infection during their lifetime [5]; however, 90% of them can eliminate the virus within two years without showing any ill effect. There is a risk for all women that HPV infections may become chronic and cause precancerous lesions, which may progress to invasive cancer (Figure 1). HPV subtypes 16 and 18 are associated with 70% of invasive cervical cancers worldwide [6]. HPVs have circular, double-stranded DNA genomes that encode eight genes, of which E6 and E7 have transforming properties and are necessary for malignant conversion. Viral proteins E6 and E7 can turn off tumor suppressor genes p53 and *pRB*, which are known to control a board range of cellular processes [7].

HPV is not the only cause of cervical cancer [8]. The proportion of cancer in cervix attributable to HPV is reported to be 83%, two-thirds of which occur in less developed countries [9]. Other risk factors such as smoking, various sexually transmitted infections (STIs) (including HIV), and immunosuppression increase the chance that women exposed to HPV are more likely to develop cervical cancer (Figure 1) [10,11]. Recent research demonstrated the role of chronic inflammation and oxidative stress (OS) in the pathogenesis of HPV infection and the associated carcinogenic processes [12]. HPV vaccines do not protect against all high-risk HPV types, and they do not treat pre-existing infections and related cervical abnormalities.

## 3. HPV Vaccination

Three vaccines, namely Cervarix (GlaxoSmithKline, UK), Gardasil, and Gardsil9 (Merck, PA, USA), were developed to prevent HPV-16- and -18-related cancers. Cervarix and the first-generation HPV vaccine, Gardasil, can prevent about 70–84% of cervical cancers [13]. A next-generation nine-valent HPV vaccine, Gardasil 9 (Merck), can prevent approximately 90% of HPV infections [14]. They are recombinant, multivalent subunit vaccines and contain virus-like particles (VLPs) derived from the L1 protein of HPV types 16 and 18. Gardasil 9 protects against infection with strains covered by the first generation of Gardasil (HPV-6, HPV-11, HPV-16, and HPV-18) and protects against five other HPV strains responsible for 20% of cervical cancers (HPV-31, HPV-33, HPV-45, HPV-52, and HPV-58).

The best time to get the vaccine is before young girls or boys become sexually active. This is because the vaccine works best before there is any chance of infection with HPV.

Over the last decade, schoolgirls across the USA and other developed countries have routinely received HPV vaccines, when they are 11–13 years old (Figure 1). Studies demonstrated that the vaccines have greatly reduced the cases of cervical precancer in young women since an immunization program was introduced about 10 years ago [15]. Of note, unvaccinated women also showed a reduction in disease, suggesting the development of “herd immunity” leads to substantial protection [16].

The Strategic Advisory Group of Experts (SAGE) of the World Health Organization (WHO) recommended a two-dose schedule of an HPV vaccine for girls below 15 years of age in the year 2014. An ongoing study from India reported that the immunogenicity in 15–18-year-old recipients with two doses of a quadrivalent HPV vaccine was noninferior to that in the 15–18-year-old recipients with three doses [17]. The comparable effectiveness of one dose to that of two or three doses of HPV vaccination has recently been reported [18]. If one-dose HPV vaccination efficacy can be confirmed in well-controlled trials, this would reduce the cost, which is the primary impediment in the introduction of vaccination programs in resource-constrained countries.

The literature suggested the effect of a vaccine lasts from six to 10 years [19]; however, long-term results are not yet certain. Although HPV vaccines can generate high titers of serum-neutralizing antibodies in animals and humans, this immunization may not be able to generate significant therapeutic effects for established or breakthrough HPV infections escaping antibody-mediated neutralization. The scope of therapeutic vaccines that may prevent the development of lesions and eliminate pre-existing lesions or malignant tumor cells need to be explored.

## 4. Screening

Screenings can detect cancer at an early stage, ensuring the treatment and follow-up of those cancers with confirmed HPV infection [20]. Various screening techniques such as visual inspection with acetic acid (VIA), visual inspection with Lugol’s iodine, visual inspection with magnification devices—magnavisualizers, Pap smears cytology, HPV DNA testing have shown efficacy (Table 1). New epigenetic tests are based on either the analysis of the DNA methylation of specific genes [21] or the measurement of the relative expression of microRNAs, which can be easily measured by RT-qPCR-based methods [22]. The applicability, advantages, and drawbacks of different screening tests are summarized in Table 1. Studies are underway to find urine-based and blood-based screening tests, which will be noninvasive and likely to be less expensive [23].

Invasive cancers are rare before the age of 30 years; screening women, who are too young, could cause adverse reactions through the detection and unnecessary treatment of spontaneously regressing low-grade lesions. The WHO recommends that countries ensure women from 30 to 49 years are screened at least once in their lifetime [24].

## 5. Cervical Cancer: The Sub-Saharan (Mali and Senegal) Africa Perspective

In Mali, cervical cancer is the most common form of cancer with the majority of infections occurring in the 15–24 years age range [25]. The incidence rate of cervical cancer in Mali is estimated to be 37.7 per 100,000 women. The prevalence of high-risk HPV ranged from 12% to 14%, and these prevalence estimates are representative of reports from other West African countries [25].

Cervical cancer happens to be the most commonly occurring form of cancer in Senegal as well; the incidence rate is comparable to that of Mali, which is 37.8 per 100,000 women [26]. Reports implicated roughly 3.2 million females aged 15 and older are at risk of developing cervical cancer [27].

Various risk factors, some of which promote HPV acquisition and others that propagate the oncogenesis of cervical cancer, are rampant in both Mali and Senegal [28]. For example, polygamy increases the chance of HPV transmission; some reports have shown that polygamy can increase this risk by at least two-fold and that the rate increases with the number of partners [29]. High parity, early pregnancies, and sexual activity at a young age are all common practices in these countries and have been shown to increase the risk of cervical cancer [28].

Malnutrition in conjunction with infections endemic to Sub-Sahara (HIV, malaria, and TB) has left the population with a compromised immune system. Reproductive tract infections are also prevalent in the region. Recent studies have implicated other STIs besides HPV with cervical cancer; Herpes simplex type 2, *Chlamydia trachomatis* and *Neisseria gonorrhea* have all been linked to an increased risk of cervical cancer [30]. Infections increase the risk of cancer through the induction of chronic inflammatory responses, which generate free radicals that promote oncogenesis.

Seventy percent of the world’s cases of HIV are diagnosed in Sub-Saharan Africa [31]. The prevalence of cervical intraepithelial neoplasia (CIN) has been estimated to be as high as 20–40% in HIV-positive women [32]. HIV-positive women are more likely to have persistent HPV infections than HIV-negative women. Hawes et al. in Senegal found invasive cervical cancer in 0.3% of HIV-negative women, compared with in 1.9% of HIV-1-positive women, 4.5% of HIV-2-positive women, and 6.9% of dually infected women [33].

There is a significant shortage of trained healthcare personnel, which affects access to health services in Sub-Saharan countries. In 2006, the WHO estimated that Africa had a needs-based shortage of 818,000 healthcare professionals (meaning doctors, nurses, and midwives) based on a country needing 2.28 healthcare professionals per 1000 population [34]. Adding to the complexity of the challenges Sub-Sahara countries face, high rates of cervical cancer, which is largely preventable, are becoming of increasing concern.

## 6. HPV Vaccination in Mali and Senegal

Anti-HPV immunization has been conducted sporadically in Mali and Senegal (mainly in urban areas); however, the short-term or long-term impact on the incidence of cervical cancer is not yet known. Ongoing immunization programs against common infectious diseases have been implemented in Mali through the support of GAVI. This program has provided Gardasil vaccine since September 2012 for girls aged 9 to 13 years. A pilot study for the introduction of HPV vaccines has been conducted in two districts, one in an urban area of Bamako and another in a rural area of the Fana district. The results of these studies are not yet available.

Similarly, the Senegalese government has also launched an HPV vaccination campaign with the support of GAVI, which started in November 2017. However, HPV vaccines are not yet widely available for the implementation of population-based vaccination programs. Few data are available regarding the prevalence and distribution of HPV types in Senegal. These epidemiological studies have potential implications for vaccination programs.

## 7. Cervical Cancer Screenings in Mali and Senegal

### 7.1. Mali

Overall, cervical cancer screenings are seldom performed in Mali. One study revealed that 82.5% of participants (n = 166) tested positive for either of the high-risk HPV types 16 or 18 [35]. The same study revealed that the co-infection with both HPV types 16 and 18 was 63% for women over 50 years of age, while co-infection was only seen in 37% of their younger counterparts [35]. Only 2.73% of participants reported ever having been screened previously [36].

Limited access to health care services has left Mali with low rates of coverage for many life-saving and preventative and curative interventions. Mali lacks economic resources and healthcare infrastructure to implement routine cervical cancer screens; these services are currently only offered at a limited number of district health centers, where physicians or midwives could provide screening by VIA upon request [37]. Treatment options (e.g., cryotherapy and electrocautery) are even sparser than screening options. Hysterectomies can only be performed at regional hospitals, and treatments for advanced disease (e.g., chemotherapy and radiation therapy) are currently unavailable in Mali. The final and arguably most significant barrier is that the women and their families are responsible for the full costs of these expensive treatments.

### 7.2. Senegal

No known population-based screening program is in progress in Senegal. Random screenings are occasionally performed in urban areas and less frequently in rural areas. It is estimated that 4.43 million (out of a population of 15.85 million) females over the age of 15 are at risk of contracting cervical tumors [38]. About 2.3% of women in the Senegalese general population are estimated to harbor cervical HPV-16/18 infections at a given time [39], and many of them will develop invasive cervical cancer.

Studies revealed an incidence rate of precancerous lesions that ranged from 5.7% to 28% in Senegal, which is comparable to a 10.1% incidence rate in similar low-income countries [40]. A cohort of Senegalese women revealed that the incidence of cervical cancer peaked between the ages of 45 and 54 years of age. Furthermore, it was revealed that the average age of sexual debut in rural areas was 16 years of age while the sexual debut for those, who screened positive for HIV, was 18.8 years [40]. In addition, 92.3% of women reported having no more than two sexual partners in their lifetime, and only 43% of them ever reported having an STI [40]. These kinds of data can be useful in the triage of the most at-risk populations and provide insight as to which screening and intervention efforts are focused on.

Like Mali, the rate of routine cervical cancer screening throughout Senegal is very low, especially among older women and those living in rural areas [41]. Only about three of 13 rural regions of Senegal have started cervical cancer screening programs, which roughly equates to only 325,000 of 1,000,000 rural Senegalese women having access to this life-saving intervention [42].

A higher proportion of sex workers in urban areas of Dakar, the capital of Senegal, is affected by HPV infection; these rates are estimated to be nearly 10 times higher than that reported among women in the general population [43]. It was recently shown that high-risk HPV genotypes affecting at least 10% of sex workers in order of decreasing frequency were HPV genotypes 52, 16, 35, 51, 33, 31, 18, and 45 [44]. It has been established that a higher number of lifetime sexual partners facilitates co-infection with multiple types of HPV and other STIs [6]. An estimated percent of 0.5% of women aged from 15 to 49 in Senegal was reported to be infected with HIV (total population of 15.85 million) [45]. Sixty-seven (15.4%) sex workers were HIV-positive, and they were significantly more affected by HPV (94% vs. 77%; *p* < 0.01) than seronegative sex workers [44]. Clearly, a high burden of HPV infection is seen in sex workers with a high frequency of coinfection with HIV and multiple HPV genotypes.

## 8. Insights and Potential Measures to Eliminate Cervical Cancer in Mali and Senegal

Cervical cancer is not yet recognized as an important public health problem in Sub-Saharan countries. Promoting awareness regarding causes and potential remedies should be considered as a critical component of any program tackling cervical cancer (Table 2). Studies have shown poor knowledge of the disease in Africa, which even cuts across different literacy levels [46]. Poor knowledge is not limited to patients alone in Africa, as health care workers, who are supposed to be better informed, do not have good knowledge of the disease either.

An important lesson learned from HIV–AIDS responses is that civil society and communities have to be at the center of humanitarian responses. Community meetings and considerations of local customs could be helpful prior to undertaking vaccination and cancer screening; this process would demonstrate support from community leaders and provide a safe space for community members to ask questions and gain a greater understanding about the purposes of these procedures [47]. The development of trust between health care workers and affected communities is crucial to the success of these humanitarian responses (Table 2). Considering the early age of first pregnancy, educational programs should start at the primary school level to sensitize young girls to the risk factors of cervical cancer. Community leaders and civil society could end stigma and discrimination. A smart investment will be to integrate cervical cancer screening and treatment services into HIV and sexual and reproductive health services if already existent [48]. Community-participatory health services policies need to be developed; such efforts would include mass cervical cancer awareness and education campaigns conducted by community health workers and skilled birth attendants on the purpose, process, and timing of cervical cancer screenings (Table 2).

The Extended Programs for Immunization (EPI) were introduced into many low-resource countries, and the EPI is believed to save 3 million young lives per year in Africa. The integration of anti-HPV vaccines into the EPI is likely to increase this number by preventing thousands of deaths from cervical cancer yearly (Table 2). An effective strategy could be to focus vaccination efforts on sex workers and women with HIV infection; this could serve to prevent further transmission of HPV through male partners to other women. In general, anti-HPV vaccination rates are low amongst young women living in rural areas. Targeting those young women for catch-up vaccinations may also be an important strategy for the prevention of cancer.

It is important to strengthen local cervical cancer screening capacity and raise national-level policy stakeholder awareness in Sub-Saharan countries. The introduction of combinational VIA and HPV DNA testing for cervical cancer screening needs to be considered. Both testing permits self-sampling with VIA being easy to perform, and once lab facilities are created for HPV testing, several samples can be analyzed in a single day. VIA may be used as an approach for a population-level epidemiological prevalence study in areas, where other screening modalities are rarely available or are too expensive [49]. Organizations like Medicine Sans Frontier, who have valuable field experience in these countries, could be implicated in implementing screening programs.

The literature suggested that self-sampling is just as effective as clinician-obtained samples and has a high rate of acceptance among women [50,51]. A study was performed in two villages of Central Senegal to assess the acceptance of self-sampling and the incidence of high-risk HPV. Samples were collected from 133 women; 98.5% of the participants felt comfortable with the self-sampling technique, 99.2% of the participants used the device correctly, and 100% of the samples collected were of good quality [41]. Furthermore, 10.5% of the samples were tested positive for HPV (high-risk HPV: 6%; low-risk HPV: 4%).

Due to the lack of routine screenings, most women in these resource-constrained countries are present with such an advanced disease, and the only suitable treatment options are palliation. In most Sub-Saharan countries, palliative treatments are extremely limited. For instance, oral morphine is only available in 11 African countries [52]. Those, who are fortunate enough to access treatment, are usually treated with radiation therapy. Radiation facilities are often nonfunctional or poorly maintained and can only be found in tertiary institutions or in the private sector [53]. Recent research into “screen and treat” programs implemented in South Africa and India showed that HPV-based screening coupled with cryotherapy treatment can have a significant impact on the reduction of the incidence of cervical cancer precursors and cervical cancer itself in low-income countries [54,55].

The authors of this article (while onsite visits) found that the cost of HPV vaccination and screening tests such as Pap tests (if available in private clinics) is exceedingly high in Mali and Senegal and thus is beyond the reach of the general population. The yearly GDPs per capita in Senegal and Mali are $1546.50 and $901, respectively. Gardasil has been approved by Malian Food and Drug Administration since 2008 but is still not available in the public health sector. Both Cerverix and Gardasil are only available in a few private pharmacies in these countries and are not affordable even to middle-class Malian or Senegalese individuals. The retail prices for Gardasil and Cervarix are as high as US$150–$190 per dose [56]. Merck and GlaxoSmithKline (GSK) claim their prices to GAVI equal their manufacturing costs (~$4.50) [56]. Given the fixed gain from annual sales in affluent markets, Merck’s break-even price to GAVI should be much lower than $4.50 [56].

Clearly, cost poses the most significant barrier to low-income countries for developing broadly available HPV immunization and cancer screening services; negotiating vaccine prices with vaccine manufactures could be one way to address this barrier. For example, Merck has a patient assistance program in the USA for individuals, who do not have health insurance; in Rwanda, this vaccine manufacturer has already agreed to supply vaccines at a lower price, demonstrating that such negotiations are indeed feasible.

In the last decade, there has been significant growth in the capabilities of suppliers and vaccine manufacturers located in developing countries for various other vaccines; however, this trend did not include HPV vaccines [57]. Opportunities exist for manufacturers to invest in HPV vaccine production for developing countries, which are ever expanding and emerging markets. Setting up vaccine production facilities in alliance with manufacturers and local governments in regions with proper ownership should be envisaged. Reliable vaccine formulations taking into consideration the genotypes of circulating infectious agents and production in appropriate quantities and at affordable prices are the cornerstone of developing global vaccination policies. However, ensuring optimal access and uptake also requires strong partnerships between private manufacturers, regulatory authorities, and national and international public health services.

## 9. Conclusions

Sub-Saharan countries are unduly burdened by cervical cancer. National HPV vaccination and screening programs could significantly reduce the incidence of cervical cancer. Until these programs are effectively introduced, measures to raise awareness about the prevention and risk factors implicating local communities are extremely important.

Presently, the exorbitant cost of vaccines and screening tests is beyond the reach of the majority of Sub-Saharan citizens, and in fact, cervical cancer is considered as a disease of poverty. It can be hoped that through the collective efforts between governments, communities, donors, the private sector, innovators, researchers, and pharmaceutical companies, productive synergies could save thousands of lives from a cancer that is realistically eradicable.

## Figures and Tables

**Table 1:** Screening tests for cervical cancer with applicability to low-income. counties (LIC). The recommended (USPSTF) age to begin screening is 21 years.

A. Screening Tests	B. Requirements	C. Advantages	D. Disadvantages	E. Remarks
Pap Test	Adequate laboratory facilities Qualified personnel to perform test	High specificity	Require more than one visit High cost for LIC Repeat testing to avoid false negatives	Require pelvic examination
Visual Inspection with Acetic Acid (VIA)	Short timed training of personnel to perform test	Low cost Easily implementable in LIC	Lower specificity than Pap False positives occur Repeat testing	Self-sampling is possible “Screen and treat” approach (WHO)
HPV Testing Not recommendedfor women ages 21 to 29. In this groupHPV infection common	Extensive health care infrastructure Highly skilled personnel Specialized lab facilities	High sensitivity Same day results Analyze multiple samples simultaneously	Risk of over diagnosis False positives occur	Self-sampling is possible HPV genotyping

A. Screening tests, B. Requirements of each test, C. Advantages of each test, D. disadvantages of each test; E. Observations and remarks.

**Table 2:** Optimization of solutions to eliminate cervical cancer in Sub-Saharan Africa.

A. Awareness	B. Vaccination	C. Screening	D. Health Care Delivery
Knowledge of causes and potential remedies	Integration of anti-HPV vaccines into EPI by participation of local government and vaccine alliance (GAVI)	Integrate regular screenings into existing HIV or other reproductive health services if available	Training local health care personnel including midwives at the primary and tertiary level
Involve civil society and communities	Immunize with 2-dose vaccine, Gardasil 4, 9 (Merck) or Cervarix (GSK) Assess vaccine efficacity in individual country, monitor post-vaccination adverse side effects if any	Educate on timing and purpose of screenings to increase participation	Local health workers will lead educational campaigns
Community meetings and respect of local customs	Trusted source (CDC) recommends that preteens receive the vaccine at around age 11 or 12 years old	Strengthen local cervical cancer screening capacity	Establish community-participatory health services
Education of disease risk factors at primary school level	Catch-up vaccinations, Target sex workers and unvaccinated women	Negotiate affordably priced screening tests	Staging of cancer Access to Cryotherapy to destroy precancerous cells on cervix, a non-expensive procedure
Community leaders help end stigmas associated with STDs and promote vaccine acceptance	Negotiate vaccine pricing with the manufactures by local government and GAVI to increase access	Adapt screening tools based on cultural and practical considerations (acceptability, feasibility, cost, etc.)	Access to surgery to remove cancerous cells Access to Radiation therapy often offered in combination with chemotherapy. Above procedures hardly practiced in Sub-Saharan Africa because of cost and lack of qualified personnel
Leverage local newspapers, journals, television, social media, and mobile phones to promote educational materials	Set up regional vaccine production plants through cooperation of manufacturing pharmaceutical companies in conjunction with government and international aid organizations	Create local infrastructure to perform various screening tests	Make available Palliative care for advanced disease

A. Awareness, B. Vaccination, C. Screening, D. Health care delivery.
